# Histologic and Molecular Patterns in Responders and Non-responders With Chronic-Active Antibody-Mediated Rejection in Kidney Transplants

**DOI:** 10.3389/fmed.2022.820085

**Published:** 2022-04-29

**Authors:** Onur Sazpinar, Ariana Gaspert, Daniel Sidler, Markus Rechsteiner, Thomas F. Mueller

**Affiliations:** ^1^Clinic of Nephrology, Department of Medicine, University Hospital Zürich, Zurich, Switzerland; ^2^Department of Pathology and Molecular Pathology, University Hospital Zürich, Zurich, Switzerland; ^3^Department of Nephrology and Hypertension, University Hospital Bern, Bern, Switzerland

**Keywords:** kidney transplantation, chronic-active ABMR, Banff classification, transcriptome, eGFR slope, therapy response

## Abstract

**Introduction:**

There is no proven therapy for chronic-active antibody-mediated rejection (caABMR), the major cause of late kidney allograft failure. Histological and molecular patterns associated with possible therapy responsiveness are not known.

**Methods:**

Based on rigorous selection criteria this single center, retrospective study identified 16 out of 1027 consecutive kidney transplant biopsies taken between 2008 and 2016 with pure, unquestionable caABMR, without other pathologic features. The change in estimated GFR pre- and post-biopsy/treatment were utilized to differentiate subjects into responders and non-responders. Gene sets reflecting active immune processes of caABMR were defined *a priori*, including endothelial, inflammatory, cellular, interferon gamma (IFNg) and calcineurin inhibitor (CNI) related-genes based on the literature. Transcript measurements were performed in RNA extracted from stored, formalin-fixed, paraffin-embedded (FFPE) samples using NanoString*™* technology. Histology and gene expression patterns of responders and non-responders were compared.

**Results:**

A reductionist approach applying very tight criteria to identify caABMR and treatment response excluded the vast majority of clinical ABMR cases. Only 16 out of 139 cases with a written diagnosis of chronic rejection fulfilled the caABMR criteria. Histological associations with therapy response included a lower peritubular capillaritis score (*p* = 0.028) along with less glomerulitis. In contrast, no single gene discriminated responders from non-responders. Activated genes associated with NK cells and endothelial cells suggested lack of treatment response.

**Conclusion:**

In caABMR active microvascular injury, in particular peritubular capillaritis, differentiates treatment responders from non-responders. Transcriptome changes in NK cell and endothelial cell associated genes may further help to identify treatment response. Future prospective studies will be needed which include more subjects, who receive standardized treatment protocols to identify biomarkers for treatment response.

**Clinical Trial Registration:**

[ClinicalTrials.gov], identifier [NCT03430414].

## Introduction

Antibody-mediated rejection (ABMR) is the major cause of late kidney allograft failure (1). Early transplant survival rates have significantly improved over the last decades, in particular due to advances in human leukocyte antigen (HLA) matching and immunosuppression leading to a significant decrease in T-cell mediated rejections (TCMR) and acute ABMRs. However, for chronic rejection processes mediated by anti-HLA antibodies effective treatments are missing. The diagnosis of chronic-active ABMR (caABMR) is most likely associated with a progressive decrease in allograft function leading to near certain transplant failure (2).

The histology-based diagnosis of transplant glomerulopathy with microvascular inflammation together with donor-specific antibodies (DSA) is frequently seen as “kiss of death” for the kidney transplant. The lack of proven, effective therapies causing either a nihilistic approach, i.e., not changing therapy to avoid side-effects of over-immunosuppression, passively monitoring the progressive decline in function or a trial of various rejection therapies with anecdotal cases in mind of functional and morphological improvements (3).

Hence, the diagnosis of caABMR leaves the clinician (and the patient) with a profound uncertainty both in regard whether to treat at all, i.e., which cases are likely to respond to therapy and with which therapy.

The identification of prognostic features of potential treatment responsiveness is needed to justify and guide treatment. Key hurdles to identify these biomarkers are the often ambiguous, heterogeneous cases and diagnoses of caABMR impacted by a multitude of parallel disease processes (4, 5). This is further complicated by the lack of solid criteria for treatment response *vs.* non-response (6), the heterogeneity of treatment approaches (3, 7–10) and the dynamic of immune-mediated injury and response not being captured by histopathology alone. Molecular profiling might detect changes not seen by morphology or clinical markers (11–16).

On this background, we decided to identify features of cases of caABMR that responded and did not respond to therapy based on a rigorous, highly “puristic” approach. The selection of cases was seen as critical, i.e., only pure, unquestionable cases of caABMR with sufficiently documented pre- and post-biopsy courses and treatment responses were chosen. This highly selective, “cherry picking” approach, however, excluded the vast majority of clinical ABMR cases, in particular those with likely ongoing other pathology processes such as glomerulonephritis, TCMR, viral infections, cases suspicious but not definite for ABMR according to Banff criteria, early rejections, incomplete clinical or laboratory data, repeat biopsies or cases without change in immunosuppressive treatment.

Stored tissue samples of these highly selected cases of treated, pure caABMR with pre-defined response criteria were processed and analyzed according to their transcript expression profiles. We hypothesized that transcript changes of an *a priori* defined set of genes, reflecting the active immune processes of ABMR, might better identify ABMR cases that improve on treatment to those that do not respond. The gene selection was literature-based and focused on genes related to endothelial function, natural killer (NK) cells, and inflammatory processes (16–18).

The objective of our study is to identify in a retrospective analysis features that differentiate caABMR treatment responders from non-responders defined by a significant treatment-associated change in the estimated glomerular filtration rate (eGFR) slope.

## Materials and Methods

### Sample Cohort

The study was approved by the cantonal ethics committee (KEC, BASEC number 2017-02130) of Zurich. This retrospective, observational, longitudinal cohort study reviewed all biopsies performed in kidney transplant patients at the University Hospital of Zurich between 01.01.2008 and 31.12.2016 with histologically confirmed caABMR (based on the Banff 2017 classification) (19).

Follow-up data included serum creatinine, proteinuria (assessed by protein/creatinine ratio in spot urine expressed in g/mmol), donor-specific HLA antibody development, medication use, level of immunosuppression, date of transplantation, date of biopsy, treatment received post-biopsy. In addition, data on age, gender, primary kidney disease, and deceased or living kidney transplant were also collected. Exclusion criteria were: age at transplantation <18 years, combined organ transplantation (incl. kidney-pancreas, kidney-liver), incomplete laboratory and/or clinical data, recurrence of the initial disease, insufficient biopsy material for transcript analysis, and documented refusal of data analysis for research purpose.

### Immunosuppressive Therapy Regimens

The baseline, maintenance immunosuppression of our patients consisted of a calcineurin inhibitor (CNI; cyclosporine or tacrolimus), an anti-proliferative agent (mycophenolic acid or azathioprine), and in some cases prednisone. Induction therapy was done with either basiliximab or anti-thymocyte globulin.

Treatment of ABMR was not standardized and based on an increase in immunosuppression. The lack of standardization is reflected in the variety and combinations of treatments applied: dose increase ± drug conversion (from cyclosporine to tacrolimus, azathioprine to mycophenolic acid) ± addition of steroid bolus, immunoadsorption, plasmapheresis, intravenous immunoglobulins (5, 10, 20–23), rituximab, and/or bortezomib (24).

All rejection therapies given within 2 months post-biopsy were recorded and classified into nine different regimens. A single patient may have been treated with more than one therapy regimen.

### Classification and Selection of the Primary Set of “139 Antibody-Mediated Rejection Biopsies”

**FIGURE 1:**
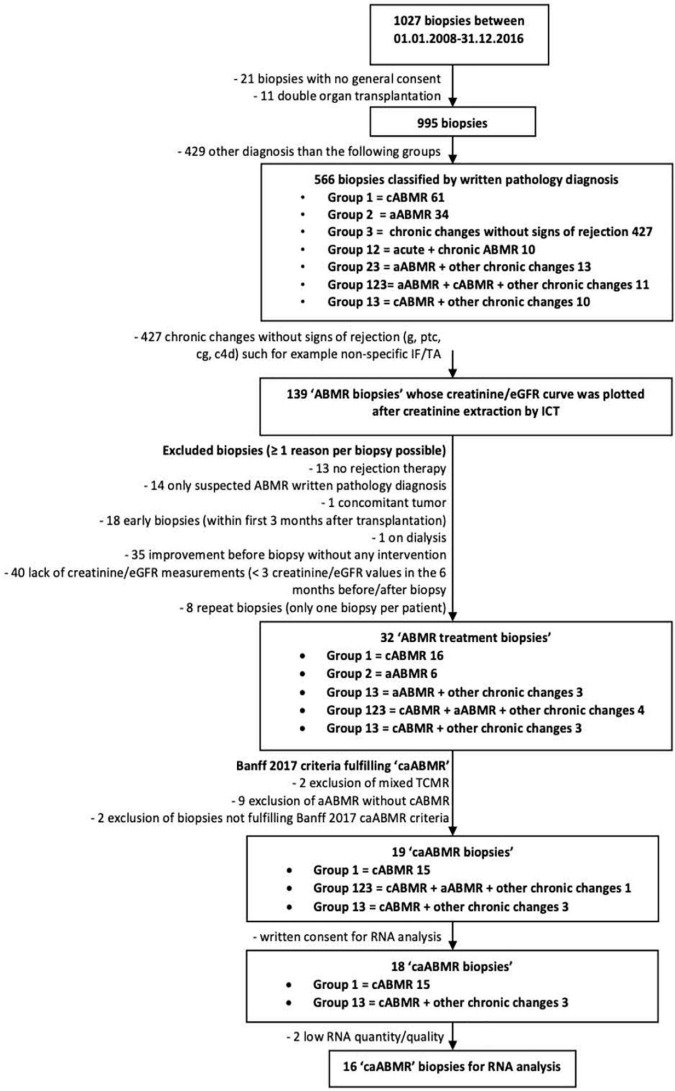
Algorithm of the biopsy selection process. cABMR, chronic antibody mediated rejection; aABMR, active antibody mediated rejection; IF/TA, interstitial fibrosis and tubular atrophy; ICT, information and communication technology; caABMR, chronic-active antibody mediated rejection fulfilling Banff 2017 criteria; ptc, peritubular capillaritis; g, glomerulitis; cg, transplant glomerulopathy; C4d, complement split product.

All transplant kidney biopsies, performed between 01.01.2008 and 31.12.2016, were pre-screened (*n* = 1027) and in a step-wise selection process the final set of biopsies was identified (see Figure 1).

In a first step biopsies of patients who did not give a general research consent (*n* = 21) and biopsies of patients with combined organ transplantation (*n* = 11, of those 8 kidney-pancreas and 3 kidney-liver transplantations) were excluded (*n* = 32 in total).

In a second step the remaining 995 biopsies were classified in 6 groups according to the written pathology diagnosis. The selection based on “descriptive words” rather than scores was chosen because in only a fraction of biopsies, taken over this extended period of time, Banff scores were available. In addition the Banff classification has changed over time.

Group 1 included all biopsies with chronic ABMR only (*n* = 61), group 2 all biopsies with active ABMR only (*n* = 34), and group 3 all biopsies with chronic changes only (such as non-specific interstitial fibrosis and tubular atrophy) without signs of ABMR (*n* = 427). Patients with biopsy diagnoses stating both chronic and active ABMR changes were included in group 12 (*n* = 10), those with both active ABMR and other chronic changes were included in group 23 (*n* = 13), those with both chronic ABMR changes and other chronic changes in group 13 (*n* = 10). Some biopsy diagnoses stated active ABMR and chronic ABMR and other chronic changes at the same time, these biopsies were included in group 123 (*n* = 11). All biopsies which had another diagnosis and could not be included in one of the groups above were excluded (*n* = 429).

Following these selection criteria a total of 139 “ABMR biopsies” were identified based on their descriptive diagnosis in words, i.e., described as either active and/or chronic ABMR (groups 1, 2, 12, 13, 123, 23). All 109 patients of these 139 “ABMR biopsies” were contacted and asked for consent.

### Identification of Therapy Responders and Non-responders

The individual serum creatinine and eGFR (25) slopes before and after these 139 biopsies were plotted to identify clinical therapy responders and non-responders. Through linear regression analysis based on the Mitch curve the trajectory of the curves 6 months before and 6 months after the biopsy time point were computed. Therapy response was defined as a slower rate of loss or a gain in eGFR from the pre- to post-biopsy/treatment periods. Treatment non-response was defined as no change or a more rapid loss of eGFR from the pre- to post-biopsy/treatment periods.

### Selection of Cases of “32 Antibody-Mediated Rejection Treatment Biopsies”

In the third selection step all 139 “ABMR biopsies” with a written diagnosis of “ABMR” were analyzed to identify those fulfilling the criteria for evaluation of treatment response or non-response. Altogether 107 biopsies were excluded, some had more than one of the exclusion criteria:

• No rejection therapy, i.e., no documented increase or addition of immunosuppressive treatment at time of biopsy (*n* = 13 biopsies).
• Suspected ABMR, i.e., biopsies that did not qualify as full picture of ABMR according to Banff criteria in the written pathology diagnosis (*n* = 14).
• Concomitant tumor, i.e., biopsy in a patient with a tumor that impacted the treatment decision (*n* = 1).
• Early biopsies, i.e., biopsies taken within the first 3 post-transplant months (*n* = 18).
• On dialysis, i.e., biopsy taken while the patient was already on dialysis (*n* = 1).
• Improvement before biopsy, i.e., cases with improvement in kidney function before the biopsy was taken and without any rejection therapy (*n* = 35).
• Lack of creatinine/eGFR measurements, i.e., biopsy cases that did not have at least 3 creatinines/eGFRs measured in each period, the 6 months before and 6 months after the biopsy, respectively (*n* = 40).
• Repeat biopsies, i.e., only one biopsy per patient was selected to avoid overlaps and putting too much weight on a single patient case (*n* = 8).

After this step a total set of 32 “ABMR treatment biopsies” met all the inclusion criteria.

### Scoring and Selection of the Final Set of “16 Chronic-Active Antibody-Mediated Rejection Biopsy Cases”

In the last selection step the set of 32 “ABMR treatment biopsies” were reread by our nephropathologist to be scored according to the definitions of the Banff 2017 classification (19). Biopsies displaying diagnostic features of either a mixed rejection phenotype (TCMR and ABMR), an active ABMR process without the chronic component, clear signs of a *de novo* or recurrent glomerulonephritis or cases in which patients did not specifically consent to transcriptome studies (6 patients out of 32) were excluded. After this rigorous selection step a remaining set of 18 biopsies showed the three diagnostic criteria of caABMR, i.e., ABMR chronicity, antibody interaction, and DSA ± C4d staining. As in two biopsy cases not enough FFPE tissue was left over for high quality RNA-processing the final set consisted of “16 caABMR cases” for transcriptome measurements (see also Figure 1).

### Gene Selection for Transcriptome Measurements in Chronic-Active Antibody-Mediated Rejection Cases

Genes of interest were selected *a priori* based on the known molecular immunopathology of the antibody-mediated rejection process. The diagnostic hallmark of ABMR is the injury to the microcirculation (26). The endothelium is presumed to be the primary target of antibodies leading to cell injury associated with pathways and signals of inflammation (18, 27–29), migration of myeloid cells and NK cells (17, 18, 27, 30–32) and interferon gamma (IFNg) related injury responses (27, 33). In addition, genes associated with calcineurin inhibitor (CNI) toxicity, that might impact chronicity changes, were also chosen. A total of 44 target genes, representing key pathways and structures associated with ABMR, and 4 house-keeping genes, were selected. Grouping and in detail description of the individual genes is summarized below and in Supplementary Table 1:

Group 1–Endothelium-associated genes: CDH5, CDH13, COL13A1, DARC, ECSCR, GNG11, ICAM2, MALL, PECAM1, PGM5, RAMP3, RAPGEF5, ROBO4, TM4SF18, VWF, THBD, SELE, PLAT, and TEK.
Group 2–Inflammation-associated genes: OSM, OSMR, SAA1, IL6, IL6R, HMGB1, CLEC4E, and IL1B.
Group 3–Cellular response-associated genes: myeloid cells KLF4, PPM1F, NK cells CCL4c, CD160, YME1L1, FGFBP2, GNLY, CX3CR1, KLRD1, KLRF1, SH2D1B, and TRDV3.
Group 4–Interferon-gamma inducible genes: CXCL10, CXCL11, and PLA1A.
Group 5–Calcineurin inhibitor toxicity: TNFSF12 and TNFRSF12A.
Group 6–House-keeping genes: ACTB, LDHA, HPRT1, and GAPDH.

### Transcriptome Measurements of the Formalin-Fixed, Paraffin-Embedded Samples of Chronic-Active Antibody-Mediated Rejection Cases

To obtain high quality transcriptome measurements in stored FFPE tissues we chose the NanoString*™* technology that allows robust mRNA analysis without further amplification (34). For NanoString*™* analysis around 100 ng total RNA is needed, i.e., 3–4 cuts of 20 um each from the FFPE block. After deparaffinization RNA was extracted and its quality checked. Based on the target mRNA sequence two specially designed oligonucleotides A and B were synthesized for each one of the 48 target mRNAs. Oligonucleotide A specifically binds the target mRNA sequence and a unique fluorescent barcode reporter specific for the target mRNA sequence. Oligonucleotide B specifically binds the target mRNA sequence and a universal capture tag able to fix the target mRNA on a plate. Forty-eight target-mRNA-specific oligonucleotides A_1,2,0.48_ and oligonucleotides B_1,2,0.48_ were synthesized by Integrated DNA Technologies*™*. Each 7 μL of the oligonucleotides A (present at 0.6 nM) and B (present at 3 nM) were mixed with 70 μL containing the unique fluorescent “barcode” reporter tag and the universal capture tag (provided by NanoString*™*), generating a so called Master Mix*™*. For hybridization 8 μL of the Master Mix*™* were added to 7 μL of FFPE RNA sample with subsequent incubation at 67°C for minimum 16 h. In a purification process all the mRNA/oligonucleotides not bound to a plate (through the universal capture tag) were washed away. After an alignment of all the hybridized unique fluorescent barcodes bound to the target mRNA in an electromagnetic field, analysis followed: the number of mRNA copies bound to their unique fluorescent barcode was counted.

### Statistics

Numerical variables were described with the mean ± standard deviation (SD). Characteristics of responders and non-responders were compared with *t*-tests for numerical variables and Chi-square-tests for categorical variables. For all calculations, *p*-values less than 0.05 were considered significant. The creatinine/eGFR curves were approximated with linear regression analysis. Data was analyzed using JASP version 0.10.2, Excel version 15.38, and XLSTAT version 20.2. Heatmap analysis of gene expression was performed with the nSolver NanoString*™* Analysis Software Version 4.0.70. Principal component analyses and hierarchical clustering was computed with XLSTAT version 20.2.

## Results

### Identification of Biopsies With a “Pure” Histological Diagnosis of Chronic-Active Antibody-Mediated Rejection and a Treatment Response That Could Be Classified

As shown in Figure 1 a total of 139 (13.5%) out of the 1027 kidney transplant biopsies collected had a written pathology report including antibody mediated rejection. From these “ABMR biopsies” those with incomplete creatinine measurements, improvement of kidney function already before biopsy, a diagnosis of only suspected ABMR, biopsies taken in the first 3 post-transplant months, and biopsies with no rejection treatment were excluded. This reduced the set to 32 “ABMR treatment biopsies.” These biopsies were reread and classified according to the Banff 2017 criteria and those with mixed TCMR/ABMR or active ABMR phenotype, low quantity or quality of FFPE sample material and lack of consent to use stored tissue were excluded and left a final set of 16 pure “caABMR” biopsies for transcriptome analysis (Figure 1).

### Patient Characteristics

The baseline characteristics of the 16 patients and their biopsies with the diagnosis of “caABMR” are given in Table 1. Demographics between the groups of 6 non-responders and 10 responders were similar. The recipients had a mean age of 44 years at transplantation and 52 years at time of biopsy; 5 (31%) were female and most kidneys were from deceased donors (63%). Biopsies were taken at a mean of 7.9 years post-transplantation, slightly but not significantly later in the responder group compared to the non-responder group (8.8 vs. 6.3 years post-transplantation, resp.). In 11 patients (69%) donor specific anti-HLA antibodies (DSA), preformed and/or *de novo*, with a median fluorescence intensity of >1000 were detected. There was no statistical difference in the total number of DSA or the cumulative total MFI between responders and non-responders, however, there was a trend toward higher DSAs and MFIs in the non-responders (data not shown). The treatments of the ABMR showed a significant heterogeneity without a clear pattern (Table 2).

**TABLE 1:** Baseline characteristics.

	Overall	Non-responder	Responder	*P*-value
Number	16	6	10	
**Demographics**				
Gender (female)	5 (31)	2 (33)	3 (30)	0.889
Age at transplantation [yrs]	44 ± 14	46 ± 18	43 ± 11	0.673
Age at biopsy [yrs]	52 ± 12	53 ± 16	52 ± 10	0.944
Age of transplant kidney at time of biopsy [yrs]	55 ± 15	57 ± 18	54 ± 14	0.693
Post-operative time of biopsy [yrs]	7.9 ± 5.5	6.3 ± 4.2	8.8 ± 6.2	0.408
Donor type (deceased donor)	10 (63)	4 (67)	6 (60)	0.790
Graft survival [yrs]		3.15 ± 2.9	4.0 ± 4.1	0.64
DSA status (positive)	11 (69)	4 (67)	7 (70)	0.889
**Written pathology diagnosis**				
cABMR	13 (81)	5 (83)	8 (80)	
cABMR + other chronic changes	3 (19)	1 (17)	2 (20)	

*Donor specific antigen (DSA) status was considered positive in individuals with preformed and/or de novo DSA with median fluorescence intensity (MFI) > 1000. Values are given as mean ± SD, or as absolute counts (percentage). Characteristics of responders and non-responders were compared with t-tests for numerical variables and Chi-square-tests for categorical variables.*

**TABLE 2:** Summary of the antibody-mediated rejection therapies in the non-responder and responder groups.

Gender	Non-responder	Responder
Increase in dose >20%	3	0
Switch Cyclosporine to Tacrolimus	4	3
Switch Azathioprine to Mycophenolate	0	3
Switch Everolimus to Mycophenolate	1	0
Steroid pulse	5	8
Immunoadsorption	1	0
Plasmapheresis	0	2
IVIG	2	6
Rituximab	0	3
Bortezomib	1	0

*IVIG, intravenous immunoglobulin.*

### Kidney Transplant Function Before and After Biopsy

**FIGURE 2:**
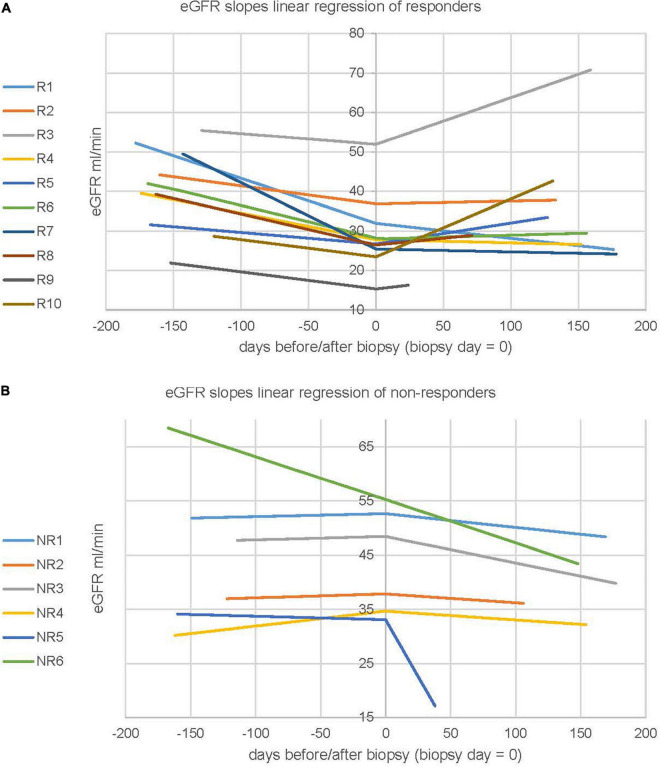
Kidney function before and after biopsy in treatment responders versus non-responders. **(A)** eGFR slopes in 10 therapy responders, **(B)** in 6 non-responders. Every line represents one patient. The linearized slopes were calculated by linear regression of raw data before and after biopsy and plotting of the two curves y_*pre–biopsy*_ = m_pre–biopsy_ *x + q_pre–biopsy_ and y = m_post–biopsy_*x + q_post–biopsy_ where q_pre–biopsy_ = q_post–biopsy_ = measured eGFR at biopsy day.

The individual slopes of kidney function, based on linear regression of at least 3 eGFR values before and 3 after biopsy, are shown in Figure 2. Pre-biopsy the decrease in kidney function over 6 months was significantly greater in the responders (Figure 2A) compared to the non-responders (Figure 2B), showing a mean slope of a −0.070 vs. −0.005 ml/min × d, resp. (*p* = 0.010). Post-biopsy over 6 months the decrease in function accelerated in the non-responders and improved in the responders, showing a mean slope of −0.103 vs. 0.035 ml/min × d, resp. (*p* = 0.023). The mean difference in the slopes before to after biopsy was −0.095 vs. 0.105 ml/min × d for non-responders and responders, resp. (0.002). The detailed numbers are given in Supplementary Table 2. As shown in Table 1 the difference in graft survival did not reach statistical significance, however, there was a trend toward longer graft survival in responders compared to non-responders.

### Proteinuria Before and After Biopsy

The mean level of proteinuria pre-biopsy was 0.15 g/mmol (extrapolates to 1.5 g per day) for non-responders and 0.16 g/mmol (extrapolates to 1.6 g per day) for responders, post-biopsy 0.24 g/mmol for non-responders, and 0.2 g/mmol for responders. The levels were not significantly different between pre- vs. post-biopsy or non-responders vs. responders as shown in Supplementary Table 3.

### Banff Features of Biopsies in Responders vs. Non-responders

Table 3 shows the average Banff scores derived from the biopsies. All 16 biopsies had signs of microvascular inflammation with peritubular capillaritis (ptc 1.31 ± 1.25) and glomerulitis (g 1.79 ± 0.89). In addition they showed hyaline arteriolar changes (ah 2.31 ± 1.01 and aah 1.38 ± 1.03) and marked signs of transplant glomerulopathy (cg 2.46 ± 0.78). The scores for tubulitis (t 0.25 ± 0.45), interstitial inflammation (i 0.33 ± 0.52), intimal arteritis (v 0.07 ± 0.27), and arterial fibrous intimal thickening (cv 0.36 ± 0.75) were low. Peritubular capillaritis was significantly higher in the non-responder compared to the responder group (ptc 2.18 ± 1.17 vs. 0.80 ± 1.03, *p* = 0.028).

**TABLE 3:** Banff scores characteristics.

Banff score	Overall	Non-responder	Responder	*P*-value
**t** score	0.25 ± 0.45	0.33 ± 0.52	0.20 ± 0.42	0.582
**i** score	0.19 ± 0.43	0.33 ± 0.52	0.10 ± 0.32	0.277^a^
**ti** score	0.69 ± 0.70	0.50 ± 0.55	0.80 ± 0.79	0.428
**ptc** score	1.31 ± 1.25	**2.18** ± 1.17	**0.80** ± 1.03	**0.028**
**v** score	0.07 ± 0.27	0.17 ± 0.41	0.00 ± 0.00	^b^
**cv** score	0.36 ± 0.75	0.17 ± 0.41	0.50 ± 0.93	0.429^a^
**g** score	1.79 ± 0.89	2.20 ± 1.10	1.56 ± 0.73	0.207
**cg** score	2.46 ± 0.78	2.00 ± 0.71	2.75 ± 0.71	0.090
**mm** score	0.44 ± 0.90	0.17 ± 0.41	0.60 ± 1.08	0.365
**ci** score	0.94 ± 0.57	1.00 ± 0.63	0.90 ± 0.57	0.748
**ct** score	0.94 ± 0.57	1.00 ± 0.63	0.90 ± 0.57	0.748
**ah** score	2.31 ± 1.01	2.50 ± 1.25	2.20 ± 0.92	0.585
**aah** score	1.38 ± 1.03	1.67 ± 1.03	1.20 ± 1.03	0.396
**c4d** score	1.25 ± 1.44	1.00 ± 1.55	1.40 ± 1.43	0.607
**i-IFTA** score	1.78 ± 1.20	2.00 ± 0.00	1.71 ± 1.38	^c^

*Banff scores of responders and non-responders were compared with t-tests. Values are given as mean ± SD. Significant values are given in bold. t, tubulitis score; i, interstitial inflammation score; ti, total cortical inflammation score; ptc, peritubular capillaritis score; v, vasculitis score; cv, arterial fibrous intimal thickening score; g, glomerulitis score; cg, transplant glomerulopathy (glomerular basement membrane double contours) score; mm, mesangial matrix expansion score; ci, interstitial fibrosis score; ct, tubular atrophy; ah, arteriolar hyalinosis score; aah, hyaline arteriolar thickening score; C4d, complement split product; IFTA, interstitial fibrosis–tubular atrophy.*

*^a^Levene’s test is significant (p < 0.05) suggesting a violation of the equal variance assumption.*

*^b^Variance of the Banff score v is equal to zero after grouping on responder and non-responder.*

*^c^Number of observations < 2 for i-IFTA after grouping on responder and non-responder.*

**FIGURE 3:**
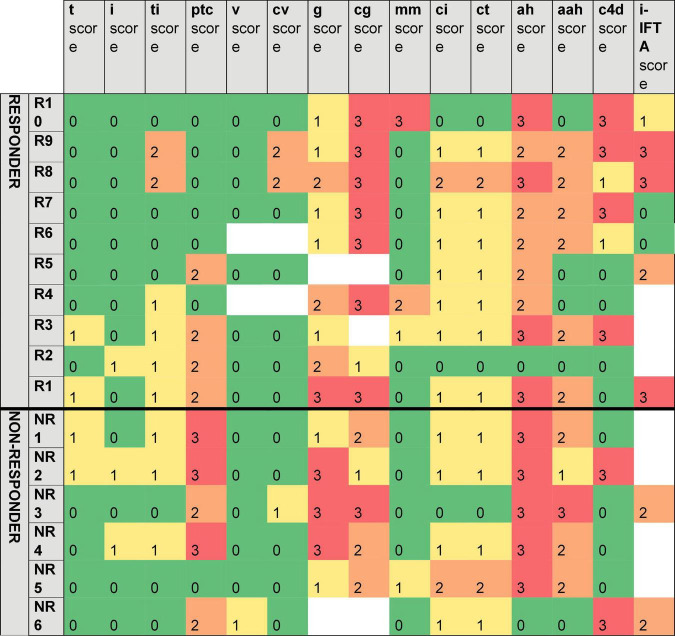
Overview of Banff scores of responders versus non-responders. t, tubulitis; i, interstitial inflammation; ti, total cortical inflammation; ptc, peritubular capillaritis; v, vasculitis; cv, arterial fibrous intimal thickening; g, glomerulitis; cg, transplant glomerulopathy; mm, mesangial matrix expansion; ci, interstitial fibrosis; ct, tubular atrophy; ah, arteriolar hyalinosis; aah, hyaline arteriolar thickening; C4d, complement split product; IFTA, interstitial fibrosis–tubular atrophy.

The heat map (Figure 3) visualizes the trends in the histopathology patterns. The non-responders show more a phenotype of active microvascular inflammation (ptc and g), whereas chronic microvascular changes characterized by glomerular basement membrane double contours were predominantly seen in the responder group.

**FIGURE 4:**
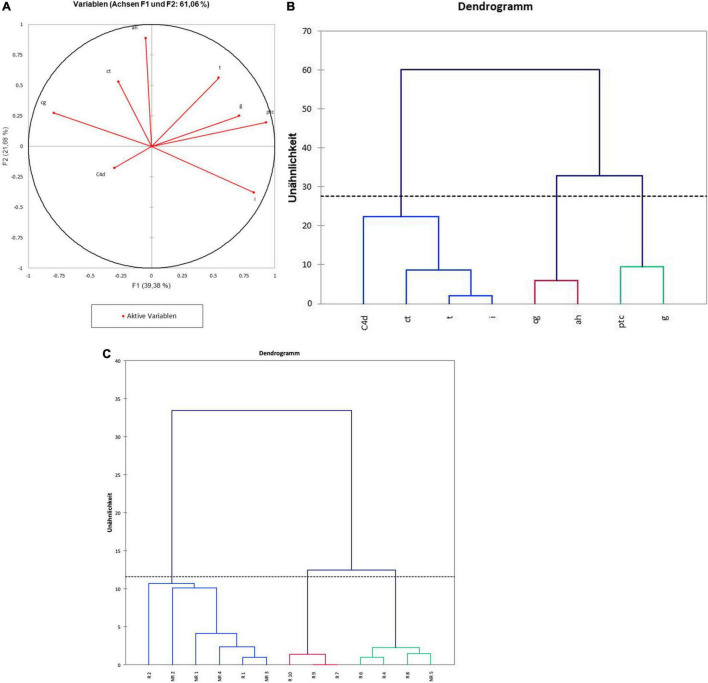
Clustering of histopathology in the caABMR biopsies. **(A)** Principal component analysis indicating distribution and correlation between Banff scores. **(B)** Unsupervised hierarchical clustering of the Banff characteristics based on the samples and **(C)** of the biopsies based on 8 Banff features. Due to high Euclidian distances 3 samples did not include in the analysis.

Overall, results of histomorphology and scoring according to the Banff 2017 criteria are shown in Figure 4A. The principal component analysis indicates that 8 Banff features explain around 61% of variability of the data. Regarding the variables ptc has the strongest weight, is positively correlated with g and to a lesser degree with t and negatively correlated with cg and not correlated with ct and ah. The x-axis differentiates acute changes (toward the right) and chronic changes (toward the left). The cluster of Banff features, shown in the dendrogram (Figure 4B), reflects the three major histological groups, active microvascular inflammation (ptc and g), chronic microvascular lesions (cg and ah) and tubulo-interstitial changes (i, t, and ct) with C4d deposition as the strongest outlier. The dendrogram in Figure 4C clusters the biopsy samples based on the profiles of 8 Banff features in the 16 biopsies. Basically, two major groups are clustered, which show a trend toward separating responders from non-responders.

### Gene Transcript Profiles in Biopsies of Responders and Non-responders

#### Transcriptome Quality and Analysis

To have sufficient sample quantity and quality in the transcriptome reading the NanoString*™* technology requires in FFPE samples a A260/280 ratio of >1.8 (protein contamination), a A260/230 ratio of >1.8 (organic contaminants), a minimal concentration of 20–60 ng/ul of RNA and at least 50% of the sample being greater than 300 nucleotides in length. Only 2 out of 18 biopsies could not be analyzed because of a too low RNA concentration, average concentrations, ratios and fragment lengths are given in Supplementary Table 4. Overall there was good RNA quality achieved and there were no significant differences between the responder and non-responder groups.

The average transcript levels and fold change of genes between responders and non-responders is given in Supplementary Table 5. The transcript levels of the 44 target genes showed a similar expression pattern in therapy responders vs. non-responders. After adjustment for multiple testing no gene was significantly differently expressed between the two therapy groups, although NK cell related genes were showing a tendency to be relatively more expressed in non-responders. The genes of each pathophysiological category showing the biggest fold change in gene expression were: VWF in the endothelial gene group (being expressed 1.6-fold in responders), CLEC4E in the inflammatory gene group (being expressed 1.7-fold in responders), CD160 in the NK cell related gene group (being expressed 2.2-fold in non-responders), and CXCL10 in the IFNg related group (being expressed 1.5-fold in non-responders). The maximal normalized fold change in gene expression between non-responders and responders was a 2.2-fold change, seen with CD160.

**FIGURE 5:**
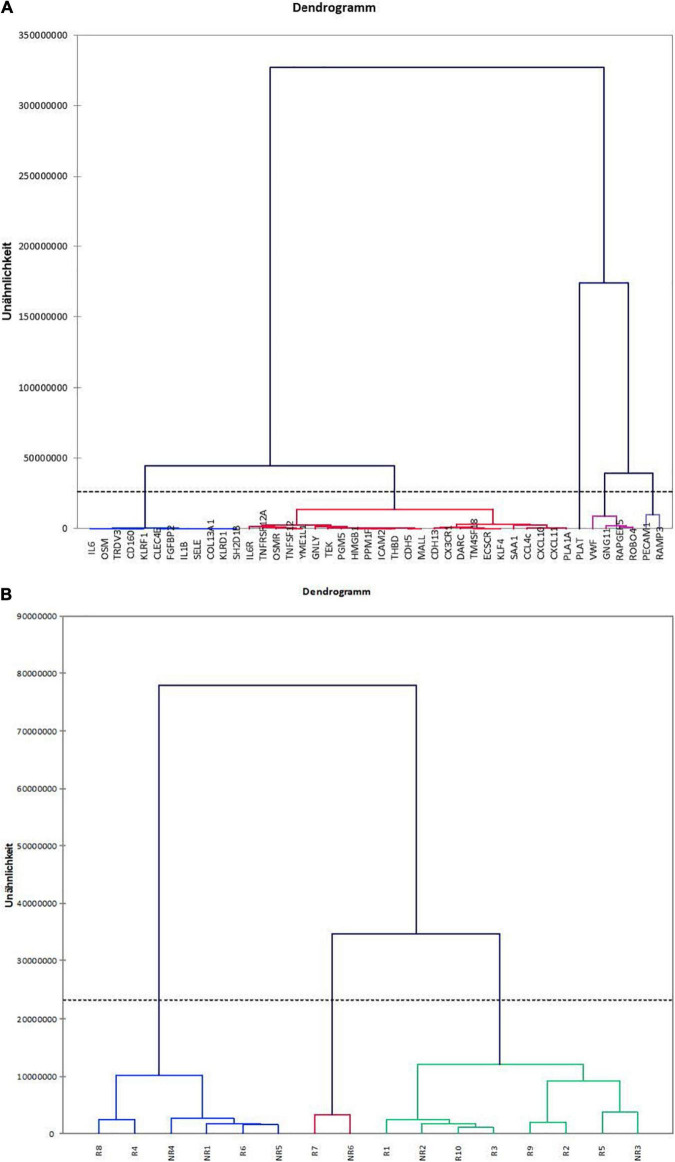
Clustering of transcriptomes in the caABMR biopsies. **(A)** Unsupervised hierarchical clustering of the 44 gene transcripts. The dendrogram shows the clustering of the 44 genes according to similarities and dissimilarities. **(B)** Dendrogram of distribution of responders and non-responders based on gene expression.

The unsupervised hierarchical clustering, regarding similarities and dissimilarities between the different genes, identified clusters that largely reflect the inflammatory and immunological pathways (Figure 5A). The dendrogram clusters genes toward pathophysiological classes with endothelial related genes being grouped together and partly separated from NK cells/inflammation related genes, which cluster together. The IFNg-inducible genes also group together. CNI toxicity related genes also order very closely. Interestingly, the two myeloid cells related genes are grouped separately from each other. OSM is known to induce IL6, the two genes appear together in the dendrogram; almost the same applies for their receptors OSMR and IL6R. The NK cell related CCL4c gene is known to be induced by IFNg and groups together with IFNg-inducible genes. The clustering supports that the selected gene groups associate with the different immunological pathways. This is not biologically surprising, however, serves to support the validity of the analysis of the RNA derived from the stored FFPE samples.

Figure 5B shows the clustering of the biopsies based on the expression patterns of the 44 *a priori* selected genes. As shown, the clusters do not separate responders from non-responders. The 16 samples are distributed randomly among the clusters.

**FIGURE 6:**
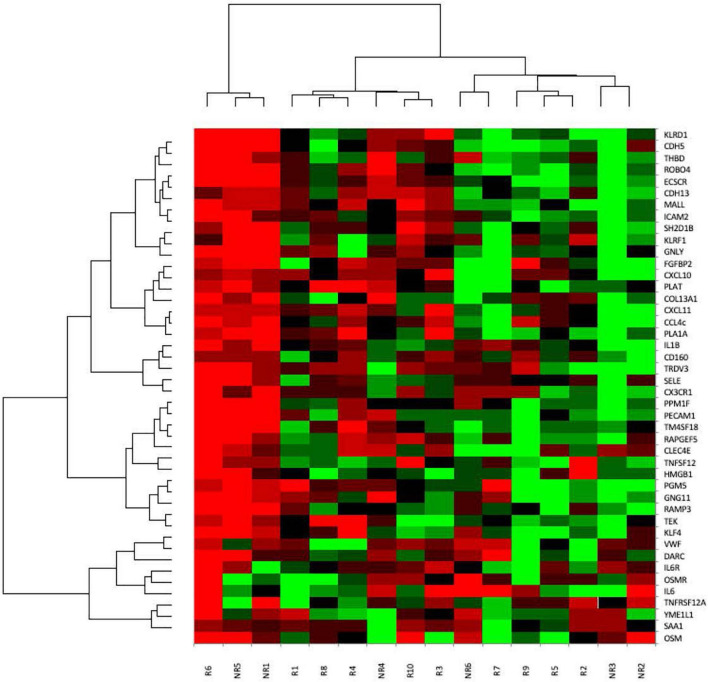
Heatmap analysis of gene expression. Columns represent the 16 different samples, rows the 44 analyzed genes. High gene expression is shown in green, low gene expression in red. Similarity metric was calculated with uncentered Pearson correlation for patients/biopsies and genes; average linkage was the used linkage method.

Gene expression was visualized through heatmap analysis (Figure 6). The similarity metric and linkage method used in the dendrogram reflects the correlation between the biopsies and genes. A trend toward higher endothelial gene expression was observed in non-responders. As expected, hierarchical clustering demonstrated that genes from the same functional category (such as endothelial, NK-cell related, etc.) behaved similarly.

## Discussion

Late allograft failure due to chronic rejection caused by donor-specific antibodies remains the key problem for allograft survival, not only for kidney but also other solid organ transplants (26). Currently, there is still no effective treatment for caABMR and the phenotype of a graft that might respond to a potential therapy is unknown (5, 35). Hence, clinically the most important question for patients with a histological diagnosis of chronic rejection is who will benefit from treatment which is not risk-free.

This retrospective study was based on the hypothesis that analysis of pure cases of caABMR, not disturbed by overlapping pathologies, is needed to identify features associated with treatment response vs. no-response. In addition, a rigorous definition of treatment response was chosen based on the trajectory of eGFR pre- and post-biopsy/treatment. Further, considering immune-mediated processes as drivers of caABMR, transcriptome measurements were added to the histopathology readings and an unbiased approach was chosen by defining gene sets *a priori*.

Due to this reductionist, very selective methodological approach, 16 “pure” caABMR cases were identified, of whom 10 responded and 6 did not respond to various therapies. Overall, active microcirculation injury, in particular histopathological severity of peritubular capillaritis, and a trend toward increased glomerulitis and transcriptome changes of activated NK and endothelial cell related genes were associated with non-response to treatment.

The rigorous selection process used here is both a strength and a weakness in that the diagnosis of ABMR was as robust as it was possible to be but the subject number was very small impacting the likelihood to detect subtle or significant changes and stratification by treatment group. The robust clinical diagnosis was based on multiple creatinine measurements before and after biopsy permitting assessment of eGFR trajectories, reclassification of the diagnosis by the pathologist according to the Banff 2017 criteria, and exclusion of cases with suspected or mixed rejection or overlapping other pathologies. In addition, the retrospective nature of the study in a single center, the lack of an untreated control group, and the heterogeneity in treatments limits any generalizability of our findings. These limitations are shared with many other ABMR treatment studies (5, 15, 36). Another inherent limitation in all ABMR studies is the lack of knowledge whether existing treatments actually work or whether clinical trajectories simply reflect the natural history of the disease in an individual patient. As AMBR remains an important clinical conundrum, we aimed to obtain as “pure” a cohort of patients as possible to reduce potential confounding. The reality is that diagnosis is challenging and there are no established treatments. Treatments are therefore individualized and in such a retrospective study as ours this is an inherent limitation that cannot be controlled for given the small numbers. We, however, felt that the likelihood of bias would be greater if we included less rigorously defined cases than if we included more cases and attempted to stratify by presence of DSA or treatment received. In this case, given the clinical importance of ABMR, we still suggest that our findings are relevant and illustrate the complexity of such a study and will permit improved planning of future studies.

The analysis of the different cases of caABMR was based on clinical markers, such as change in kidney function and degree of proteinuria, histopathology readings and gene expression profiles. Therapy response was defined by the change in eGFR slope from before compared to after therapy. In our study, kidneys with a greater degree of eGFR deterioration before biopsy were more likely to be responders, likely due to a more acute and therefore more treatable process and/or more aggressive therapy. In seeming contradiction may be the observation of more chronic changes in the glomeruli of responders. We do not have a clear explanation for this finding, however, it is known from native kidneys that impairment of GFR correlates better with tubulo-interstitial compared with glomerular injury, which may therefore not contradict our findings here. In fact, although there was a high heterogeneity in the treatment types, comparable with the literature (10, 37), antibody removal and/or rituximab were predominantly applied in the responder group (24). However, it is difficult to differentiate cause and effect. Whether presumed responsiveness lead the physician to add these treatment options or whether these therapies induced the response is in a retrospective, non-randomized analysis not possible to answer. It is unlikely that rituximab and/or plasmapheresis or a combination of rituximab with intravenous immunoglobulins are highly effective treatments of caABMR, based on the current literature [(38, 39), Triton Study by Moreso 2018]. Multiple studies indicate a lack of significant and predictable effects of these treatments (40) or any current treatment at all (41).

Proteinuria indicates kidney damage and is associated with mortality in kidney transplant patients (42–44). In our study, proteinuria showed a more pronounced increase after biopsy in non-responders compared to responders. This indicates that the injury process is more active and continuous in non-responders, in agreement with the poorer graft survival seen with proteinuria in patients with caABMR (36, 44, 45). However, the proteinuria values were quite variable across the different patients and the two treatment groups. Our findings indicate that pre-biopsy proteinuria levels are not likely indicating treatment responsiveness. As clinically expected a higher DSA burden and lower graft survival was seen in non-responders compared to responders, however, these differences did not reach statistical difference.

Interestingly, peritubular capillaritis, a histological marker of active microcirculation injury, was significantly increased in the non-responder group. The biological validity of this finding was supported by a parallel increase in glomerulitis scores, again a feature of active microvascular injury (19). This indicates that a high severity of the active endothelial injury process makes a treatment response unlikely. The degree of microvascular injury is a known risk factor for future graft loss (5, 19). In contrast, to our surprise, transplant glomerulopathy was increased in responders. This might indicate that decreased activity of the humoral immune attack and a more chronic phenotype in the vasculature reflects an exhaustion of the rejection process and a repair process in the allografts. However, transplant glomerulopathy has been associated with an increased risk of graft failure (19, 46, 47). Another explanation might be that we defined treatment response by a positive change in the GFR slope during the first 6 months post-biopsy. Hence, the improvement might reflect a transient positive anti-inflammatory effect of the rejection therapy (36). The long-term effect of transplant glomerulopathy might be negative.

Gene expression profiles did not significantly differ between responders and non-responders. However, there was a tendency toward increased endothelial and NK-cell related gene expression in non-responders compared to responders. This would fit with the histological findings of increased peritubular capillaritis. The higher peritubular capillaritis and endothelial gene expression in non-responders may capture the ongoing marked tissue injury not reversible by current ABMR therapies (17, 36, 48).

For the gene expression analysis we have selected, based on literature and pathophysiology, an *a priori* defined set of genes (16–18, 27) and some additional genes of interest. The analysis of gene expression is well-established, highly reproducible, cost-effective and technically advanced (27). However, limitations of that technology have to be considered. A difference in gene transcription does not necessarily imply a difference in protein level, the mechanisms involved in the various pathways still are not completely understood (49) and vascular or glomerular structures are underrepresented in the whole genome transcriptome profile (since 80% of the cortical tissue in the kidney is tubulo-interstitial tissue). Furthermore, the number of nephrons, the mismatch of donor recipient size, the degree of glomerulosclerosis, and the past medical history (such as diabetes and hypertension) are not likely to be fully reflected in the transcriptome and require the integration of clinical history and histopathology (14). Last but not least inter-laboratory variability and lack of powered studies in gene expression analysis also represent a problem (50).

Hence, the lack of finding significant differences of transcriptome changes in our study might be due to the intrinsic limitations of this technology. In addition, the *a priori* selection of genes might not capture all important pathway changes associated with the rejection process. Another possibility is that the measurements with NanoString*™* technology in FFPE samples, some of them of considerable age, might lack the sensitivity and specificity to detect minor changes between possible responder and non-responder profiles. Nevertheless, it was reassuring to see that the biologically defined functional gene groups clustered together in our analysis. This supports the quality of our measurements despite the considerable age of the stored biopsy samples. A major problem is the limited number of cases.

Moreover, transcriptome measurement of a single biopsy represents a snapshot of a process going on for a longer period of time and may not be sufficient. Earlier and repeated biopsies may allow to discover changes over time that reflect tissue injury and regenerative capacity and required to identify clinical responders versus non-responders.

In conclusion, a discrimination of responder vs. non-responder cases in caABMR has not yet been achieved (5, 8, 14, 15, 26, 27, 36, 43). Our results reflect the general lack of successful treatments of caABMR, partly due to the difficulty to identify possible responders from non-responders. We could show that it is possible to measure transcriptome changes in stored FFPE samples and that functional groups of genes cluster together. The rigorous selection process of clear caABMR cases indicated that the majority of biopsies are reflecting overlapping clinical diagnoses and different processes. In addition, it underlined the difficulty to classify biopsy samples based on histopathology. The identification of the severity of microcirculation injury, reflected by histological peritubular capillaritis and endothelial transcriptome activation, indicates the potential to use this for treatment guidance. However, the number of identified pure cases of caABMR in our study is too small for robust recommendations. Nevertheless, approach and results indicate key elements necessary in a future study, in particular it should be prospective, with biopsies characterized by histopathology and molecules and with rigorous functional end-points detecting response to standardized treatment protocols.

## Data Availability Statement

The original contributions presented in the study are publicly available. This data can be found here: https://www.ncbi.nlm.nih.gov/geo/query/acc.cgi?acc=GSE192552.

## Ethics Statement

The studies involving human participants were reviewed and approved by Kantonale Ethikkommission Zürich (KEK). The patients/participants provided their written informed consent to participate in this study.

## Author Contributions

OS and TM participated in design of the work, data collection, data analysis, and writing of the manuscript. OS performed the transcript measurements. AG and DS participated in writing. AG and MR participated in data collection. All authors contributed to the article and approved the submitted version.

## Conflict of Interest

The authors declare that the research was conducted in the absence of any commercial or financial relationships that could be construed as a potential conflict of interest.

## Publisher’s Note

All claims expressed in this article are solely those of the authors and do not necessarily represent those of their affiliated organizations, or those of the publisher, the editors and the reviewers. Any product that may be evaluated in this article, or claim that may be made by its manufacturer, is not guaranteed or endorsed by the publisher.
