# Supplementary material for: Histologic and Molecular Patterns in Responders and Non-responders With Chronic-Active Antibody-Mediated Rejection in Kidney Transplants
Source: Front Med (Lausanne). 2022 Apr 29;9:820085. doi: 10.3389/fmed.2022.820085 (PMC9099145; doi:10.3389/fmed.2022.820085)
Supplement: Supplementary file 1 [file Table_1.docx]

| **Group** | **Gene** | **Function** | **Paper** |
| --- | --- | --- | --- |
| **Endo-**  **thelial** | **CDH5, CDH13** | Cadherin. Calcium-dependent cell-cell adhesion molecule (in desmosoms and adherens junctions). | (18, 27, 28) |
|  | **COL13A1** | Collagen type XIII alpha 1 chain. Nonfibrillar collagen | (27) |
|  | **DARC (ACKR1)** | Duffy antigen receptor for chemokines. Promiscuous chemokine receptor and a binding protein for the malarial parasite Plasmodium vivax. | (18,27,52-54) |
|  | **ECSCR** | Endothelial cell surface expressed chemotaxis and apoptosis regulator. | (27) |
|  | **GNG11** | Guanine nucleotide-binding protein. Transmembrane signaling protein. | (27,55) |
|  | **ICAM2** | Intercellular adhesion molecule 2. Enables lymphocyte adhesion/rolling on endothelium and extravasation | (27) |
|  | **MALL** | Mal, T cell differentiation protein like. Element of the machinery for raft-mediated trafficking in endothelial cells | (18,27,28) |
|  | **PECAM1** | Platelet and endothelial cell adhesion molecule 1.  Protein found on the surface of platelets, monocytes, neutrophils, and some types of T-cells. Makes up a large portion of endothelial cell intercellular junctions; involved in leukocyte migration, angiogenesis, and integrin activation. | (18,27-29) |
|  | **PGM5** | Phosphoglucomutases. PGM activity is essential in formation of carbohydrates from glucose-6-phosphate and in formation of glucose-6-phosphate from galactose and glycogen. | (27) |
|  | **RAMP3** | Receptor (calcitonin) activity modifying proteins (RAMPs). Required to transport calcitonin-receptor-like receptor (CRLR) to the plasma membrane. | (27) |
|  | **RAPGEF5** | Rap guanine nucleotide exchange factor 5. Guanine nucleotide exchange factors (GEFs), such as RAPGEF5, serve as RAS activators by promoting acquisition of GTP to maintain the active GTP-bound state and are the key link between cell surface receptors and RAS activation. | (27) |
|  | **ROBO4** | Roundabout guidance receptor 4. Important in kidney organogenesis. Through its downstream signaling molecules, Cdc 42 and Rac 1, is involved in actin cytoskeleton remodeling and filopodia. Increased expression of Robo4 and therefore podocyte motility may result in proteinuria (in FSGS). | (18,27,56) |
|  | **TM4SF18** | Transmembrane 4 L six family member 18. Amplifies VEGF signaling and is important in angiogenesis. TM4SF18 is activated by VEGF and gives a positive feedback to VEGF. | (27,56) |
|  | **VWF** | Von Willenbrand Factor. Stored in Weibel-Palade bodies of endothelial cells and α-granules of platelets. Binds to exposed collagen (below endothelium) and to platelets. | (18,27,29) |
|  | **THBD** | Thromobomodulin. Expressed from endothelial cells it forms a thrombin-thrombomodulin complex which acts as an anticoagulant (activates protein C, which with protein S cleaves and inactivates Va, VIIIa). | (18,28,57) |
|  | **SELE** | Selectin E. Responsible for the accumulation of blood leukocytes at sites of inflammation by mediating the adhesion of cells to the vascular lining (margination and rolling of leukocytes like ICAM). | (18,28,58) |
|  | **PLAT** | Plasminogen activator, tissue. Activates plasminogen to plasmin in order to enable fibrinolysis. | (18) |
|  | **TEK** | TEK tyrosine kinase, endothelial. | (18) |
| **NK cells** | **CCL4c** | C-C motif chemokine ligand 4. Expressed in various cell types and inducible by IFNG treatment. CCL4 is expressed in T and NK cells and inducible in macrophages. | (27,32,59) |
|  | **CD160** | Binds both classical and non-classical MHC I. | (17,27,32,59) |
|  | **YME1L1** | YME1 like 1 ATPase. Plays a role in mitochondrial protein metabolism. | (17,27) |
|  | **FGFBP2** | Fibroblast growth factor binding protein 2. | (17,18,27,30,31) |
|  | **GNLY** | Granulysin. Creates holes in the target cell membrane and destroys it. | (17,18,27) |
|  | **CX3CR1** | C-X3-C motif chemokine ligand 1 (fractalkine receptor). Leukocyte recruitment in acute and chronic inflammatory. | (17,27,31,32) |
|  | **KLRD1** | Killer cell lectin like receptor D1. Regulates NK cells. | (17,27) |
|  | **KLRF1** | Killer cell lectin like receptor F1. Expressed on nearly all natural killer (NK) cells, stimulates their cytotoxicity and cytokine release. | (17,18,27,31) |
|  | **SH2D1B** | SH2 domain‐containing molecule EAT2. Regulates APCs. By binding phosphotyrosines through its free SH2 domain, EAT2 regulates signal transduction through receptors expressed on the surface of antigen-presenting cells. | (17,18,27,31) |
|  | **TRDV3** | T cell receptor delta variable 3. | (27) |
| **IFNg inducible** | **CXCL10, CXCL11** | C-X-C motif chemokine ligand 10 and 11. Stimulation of monocytes, natural killer and T-cell migration, modulation of adhesion molecule expression. | (17,27) |
|  | **PLA1A** | Phospholipase A1 member A. Signaling. | (17,18,27) |
| **Myeloid Cells** | **KLF4** | Kruppel‐like factor 4 (gut). Control the G1-to-S transition of the cell cycle following DNA damage by mediating the tumor suppressor gene p53 (unclear function in kidney). | (17,18,27) |
|  | **PPM1F** | Protein phosphatase, Mg2+/Mn2+ dependent 1F. Overexpression of this phosphatase or CAMK2G has been shown to mediate caspase-dependent apoptosis. | (27) |
| **Inflam-mation** | **OSM, OSMR** | Oncostatin M. Cytokine and growth regulator that inhibits the proliferation of a number of tumor cell lines. Also regulates the production of other cytokines, including IL 6, GCSF, GM-CSF in endothelial cells. | (60) |
|  | **SAA1** | Serum Amyloid A1. Acute phase protein highly expressed in response to inflammation and tissue injury. Also plays an important role in HDL and cholesterol homeostasis. High levels are associated with chronic inflammatory diseases including atherosclerosis, rheumatoid arthritis, Alzheimer's and Crohn's disease. | (61-63) |
|  | **IL6, IL6R** | Interleukin 6 and its receptor. Stimulates Acute Phase Protein production. Tocilizumab is an anti-IL-6 receptor drug. Excessive IL-6 production is associated with activation of T-helper 17 cell and inhibition of regulatory T cell with attendant inflammation. Plasmablast production of IL-6 is critical for initiation of T follicular helper cells and production of high-affinity IgG. | (64,65) |
|  | **HMGB1 (SBP1)** | High mobility group box 1. Key inflammatory mediator through MyD88/mitogen-activated protein kinase signaling by binding to its receptors including the receptor for advanced glycation end products or Toll-like receptors. HMGB1 plays an important role in kidney diseases, such as glomerulonephritis, lupus nephritis, antineutrophilic cytoplasmatic antibody-associated vaculitis, diabetic nephropathy, renal allograft rejection and acute kidney injury. | (65,66) |
|  | **CLEC4E (MINCLE)** | CLEC4E - C-type lectin domain family 4 member E; MINCLE macrophage-inducible C-type lectin. TLR4 ligands (Cisplatin, lipopolysaccharide, unilateral ureteric obstruction; all resulting out of acute kidney insults) induce Mincle expression in macrophages through TLR4R/NF-κB (TLR4R induces NF-κB expression). Mincle induction promotes and maintains phenotypic change to M1 macrophages (proinflammatory) via Syk signaling pathway leading to a deterioration of AKI. | (67,68) |
|  | **IL1B** | Interleukin 1 beta. Produced by activated macrophages. Important mediator of the inflammatory response, involved in cell proliferation, differentiation, and apoptosis. | (17) |
| **CNI toxicity** | **TNFSF12 (TWEAK), TNFRSF12A** | TNFS12, TNF superfamily member 12, TWEAK, TNF‐related weak inducer of apoptosis; TNF receptor superfamily member 12A. Indispensable role in the pathogenesis of acute calcineurine inhibitor toxicity lesions in mice. | (69) |
| **House-keeping** | **ACTB** | Actin beta. Involved in cell motility, structure, integrity, and intercellular signaling. | (18) |
|  | **LDHA** | Lactate dehydrogenase A. Catalyzes the conversion of L-lactate and NAD to pyruvate and NADH in the final step of anaerobic glycolysis. | (18) |
|  | **HPRT1 (HGPRT)** | Hypoxanthine phosphoribosyltransferase 1. Catalyzes conversion of hypoxanthine to IMP and GMP via transfer of the 5-phosphoribosyl group from 5-phosphoribosyl 1-pyrophosphate. Central role in the generation of purine nucleotides through the purine salvage pathway. Mutations result in Lesch-Nyhan syndrome or gout | (18) |
|  | **GAPDH** | Glyceraldehyde-3-phosphate dehydrogenase. | (18) |

**Supplementary Table 1. Gene selection**. Genes selected based on the presumed immunopathology of the antibody-mediated rejection process and their function. Gene groups selected were endothelial genes, inflammation-associated genes, cellular responses-associated genes, interferon-gamma inducible genes, calcineurin inhibitor toxicity associated genes and housekeeping genes. NK, Natural killer (cell); IFNg, interferon-gamma; CNI, calcineurin inhibitor.
