# Supplementary material for: Histologic and Molecular Patterns in Responders and Non-responders With Chronic-Active Antibody-Mediated Rejection in Kidney Transplants
Source: Front Med (Lausanne). 2022 Apr 29;9:820085. doi: 10.3389/fmed.2022.820085 (PMC9099145; doi:10.3389/fmed.2022.820085)
Supplement: Supplementary file 2 [file Table_2.docx]

| **eGFR slope** | **Overall** | **Non-Responder** | **Responder** | **P-Value** |
| --- | --- | --- | --- | --- |
| **Before** biopsy  (ml / min x day)  (ml / min x year) | -0.046 ± 0.05  -17 | -0.005 ± 0.039  -1.8 | -0.070 ± 0.044  -26 | **0.010** |
| **After** biopsy  (ml / min x day)  (ml / min x year) | -0.017 ± 0.12  -6.2 | -0.103 ± 0.157  -38 | 0.035 ± 0.059  13 | **0.023** |
| **Difference** before/after  (ml / min x day)  (ml / min x year) | 0.030 ± 0.14  12 | -0.095 ± 0.158  - 35 | 0.105 ± 0.046  38 | **0.002** |

**Supplementary Table 2. Kidney function in treatment responders and non-responders.** The eGFR slopes before and after biopsy in response to ABMR treatment are given, the slopes of responders and non-responders were compared with t-tests. Values are given as mean ± SD. Significant values are given in bold.
