# Supplementary material for: Histologic and Molecular Patterns in Responders and Non-responders With Chronic-Active Antibody-Mediated Rejection in Kidney Transplants
Source: Front Med (Lausanne). 2022 Apr 29;9:820085. doi: 10.3389/fmed.2022.820085 (PMC9099145; doi:10.3389/fmed.2022.820085)
Supplement: Supplementary file 3 [file Table_3.docx]

| **Proteinuria slope** | **Overall** | **Non-Responder** | **Responder** | **P-Value** |
| --- | --- | --- | --- | --- |
| **Before** biopsy  (g / day x day)  (g / day x year) | 0.0007 ± 0.001  0.255 | 0.001 ± 0.001  0.365 | 0.0004 ± 0.0008  0.146 | 0.094 |
| **After** biopsy  (g / day x day)  (g / day x year) | 0.00004 ± 0.002  0.0146 | -0.0002 ± 0.002  -0.073 | 0.0002 ± 0.001  0.073 | 0.667 |
| **Difference** before/after  (g / day x day)  (g / day x year) | -0.0006 ± 0.002  0.219 | -0.001 ± 0.002  -0.365 | -0.00001 ± 0.002  0.00365 | 0.200 |

**Supplementary Table 3. Proteinuria slope.** Proteinuria slope before/after biopsy and ABMR treatment. As an example, a creatinine slope of -0.0007 g/d x d represents a 0.255 g/d proteinuria increase in a year. The slopes of responders and non-responders were compared with t-tests. Values are given as mean ± SD. Significant values are given in bold.
