# Supplementary material for: Histologic and Molecular Patterns in Responders and Non-responders With Chronic-Active Antibody-Mediated Rejection in Kidney Transplants
Source: Front Med (Lausanne). 2022 Apr 29;9:820085. doi: 10.3389/fmed.2022.820085 (PMC9099145; doi:10.3389/fmed.2022.820085)
Supplement: Supplementary file 4 [file Table_4.docx]

|  | **Overall** | **Non-Responder** | **Responder** | **P-Value** |
| --- | --- | --- | --- | --- |
| **RNA concentration ng/µl** | 29.5 ± 16.4 | 35.8 ± 21.0 | 25.8 ± 12.7 | 0.249 |
| **A260/280 Ratio** | 2.19 ± 0.30 | 2.05 ± 0.11 | 2.27 ± 0.35 | 0.160 |
| **A280/230 Ratio** | 11.9 ± 33.4  2.9 (2.2; 137) | 3.25 ± 1.49  2.7 (2.2; 6.2) | 17.2 ± 42  3.0 (2.2; 137) | 0.437 |
| **% of RNA fragments with a length 50-300 nucleotides** | 18.4 ± 6.3 | 17.1 ± 8.0 | 19.1 ± 5.3 | 0.568 |

**Supplementary Table 4. RNA quantity and quality of the 16 caABMR FFPE probes whose RNA was analyzed.** Values are given as mean ± SD, median (min; max).
