# Supplementary material for: Histologic and Molecular Patterns in Responders and Non-responders With Chronic-Active Antibody-Mediated Rejection in Kidney Transplants
Source: Front Med (Lausanne). 2022 Apr 29;9:820085. doi: 10.3389/fmed.2022.820085 (PMC9099145; doi:10.3389/fmed.2022.820085)
Supplement: Supplementary file 5 [file Table_5.docx]

| **Gene** | **Pathophysiological Class** | **Overall** | **Non-Responder** | **Responder** | **Fold change** | **p-Value** |
| --- | --- | --- | --- | --- | --- | --- |
| **LDHA Control** | **CTRL** | 0.64 ± 0.20 | 0.60 ± 0.23 | 0.66 ± 0.19 | 1:1.1 | 0.596 |
| **HPRT1 Control** | **CTRL** | 0.54 ± 0.17 | 0.48 ± 0.11 | 0.58 ± 0.20 | 1:1.2 | 0.304 |
| **GAPDH Control** | **CTRL** | 0.34 ± 0.19 | 0.30 ± 0.08 | 0.37 ± 0.23 | 1:1.2 | 0.487 |
| **ACTB Control** | **CTRL** | 0.73 ± 0.16 | 0.65 ± 0.12 | 0.78 ± 0.17 | 1:1.2 | 0.124 |
| **CCL4c** | **NK** | 417 ± 305 | 472 ± 304 | 384 ± 317 | 1.2:1 | 0.595 |
| **CD160** | **NK** | 15.2 ± 18.4 | 23.2 ± 26.9 | 10.4 ± 9.9 | 2.2:1 | 0.189 |
| **CDH13** | **END** | 431 ± 292 | 525 ± 425 | 375 ± 180 | 1.4:1 | 0.338 ᵃ |
| **CDH5** | **END** | 779 ± 238 | 682 ± 272 | 837 ± 208 | 1:1.2 | 0.218 |
| **CLEC4E** | **INFL** | 109 ± 95 | 75.8 ± 77.2 | 130 ± 102 | 1:1.7 | 0.287 ᵃ |
| **COL13A1** | **END** | 103 ± 35.4 | 106 ± 47.1 | 102 ± 29 | 1:1 | 0.833 |
| **CX3CR1** | **NK** | 265 ± 170 | 326 ± 248 | 228 ± 100 | 1.4:1 | 0.281 ᵃ |
| **CXCL10** | **IFNG** | 450 ± 335 | 587 ± 445 | 368 ± 240 | 1.5:1 | 0.216 ᵃ |
| **CXCL11** | **IFNG** | 501 ± 425 | 540 ± 458 | 478 ± 428 | 1.1:1 | 0.786 |
| **DARC** | **END** | 488 ± 277 | 378 ± 152 | 554 ± 319 | 1:1.4 | 0.231 |
| **ECSCR** | **END** | 414 ± 143 | 384 ± 177 | 432 ± 126 | 1:1.1 | 0.536 |
| **FGFBP2** | **NK** | 61.4 ± 47.3 | 70.3 ± 61.1 | 56.0 ± 39.6 | 1.2:1 | 0.575 ᵃ |
| **GNG11** | **END** | 1630 ± 354 | 1573 ± 443 | 1665 ± 311 | 1:1 | 0.631 |
| **GNLY** | **NK** | 667 ± 433 | 603 ± 480 | 705 ± 425 | 1:1.1 | 0.664 |
| **HMGB1** | **INFL** | 701 ± 132 | 682 ± 109 | 712 ± 148 | 1:1 | 0.683 |
| **ICAM2** | **END** | 723 ± 193 | 749 ± 266 | 708 ± 149 | 1:1 | 0.699 |
| **IL1B** | **INFL** | 72.8 ± 39.4 | 86.8 ± 55.4 | 64.3 ± 25.9 | 1.3:1 | 0.283 ᵃ |
| **IL6** | **INFL** | 11.3 ± 9.69 | 10.3 ± 11.4 | 11.9 ± 9.11 | 1:1.1 | 0.766 |
| **IL6R** | **INFL** | 924 ± 313 | 970 ± 412 | 897 ± 259 | 1:1 | 0.668 |
| **KLF4** | **MYE** | 434 ± 121 | 392 ± 117 | 460 ± 121 | 1:1.1 | 0.291 |
| **KLRD1** | **NK** | 100 ± 54 | 93.3 ± 66.7 | 105 ± 48.4 | 1:1.1 | 0.701 |
| **KLRF1** | **NK** | 28.1 ± 14.4 | 26.5 ± 18.7 | 29.0 ± 12.2 | 1:1 | 0.750 |
| **MALL** | **END** | 833 ± 218 | 898 ± 264 | 794 ± 190 | 1.1:1 | 0.373 |
| **OSM** | **INFL** | 11.3 ± 7.49 | 8.33 ± 7.42 | 13.0 ± 7.33 | 1:1.5 | 0.240 |
| **OSMR** | **INFL** | 797 ± 216 | 781 ± 222 | 807 ± 223 | 1:1 | 0.826 |
| **PECAM1** | **END** | 3154 ± 923 | 2919 ± 967 | 3294 ± 918 | 1:1.1 | 0.451 |
| **PGM5** | **END** | 804 ± 254 | 807 ± 277 | 803 ± 256 | 1:1 | 0.977 |
| **PLA1A** | **IFNG** | 563 ± 266 | 537 ± 240 | 578 ± 292 | 1:1 | 0.778 |
| **PLAT** | **END** | 4499 ± 2609 | 4508± 3184 | 4493 ± 2391 | 1:1 | 0.992 |
| **PPM1F** | **MYE** | 788 ± 184 | 735 ± 203 | 820 ± 174 | 1:1.1 | 0.387 |
| **RAMP3** | **END** | 2251 ± 618 | 2149 ±  740 | 2312 ± 567 | 1:1 | 0.626 |
| **RAPGEF5** | **END** | 1187 ± 401 | 1132 ± 362 | 1220 ± 438 | 1:1 | 0.686 |
| **ROBO4** | **END** | 1364 ± 486 | 1291 ± 647 | 1408 ± 394 | 1:1 | 0.659 |
| **SAA1** | **INFL** | 302 ± 414 | 304 ± 440 | 301 ± 422 | 1:1 | 0.986 |
| **SELE** | **END** | 74.6 ± 39.8 | 79.3 ± 63.1 | 71.7 ± 20.1 | 1.1:1 | 0.724 ᵃ |
| **SH2D1B** | **NK** | 89.5 ± 55.1 | 97.3 ± 76.1 | 84.8 ± 42.2 | 1.1:1 | 0.675 |
| **TEK** | **END** | 665 ± 238 | 620 ± 253 | 692 ± 239 | 1:1.1 | 0.578 |
| **THBD** | **END** | 743 ± 161 | 701 ± 217 | 768 ± 123 | 1:1 | 0.435 |
| **TM4SF18** | **END** | 526 ± 144 | 509 ± 166 | 537 ± 139 | 1:1 | 0.716 |
| **TNFRSF12A** | **CNI** | 802 ± 295 | 763 ± 275 | 826 ± 318 | 1:1 | 0.696 |
| **TNFSF12** | **CNI** | 896 ± 201 | 914 ± 124 | 886 ± 241 | 1:1 | 0.800 |
| **TRDV3** | **NK** | 28.3 ± 27.1 | 39.2 ± 32.3 | 21.7 ± 22.8 | 1.8:1 | 0.224 |
| **VWF** | **END** | 1491 ± 941 | 1058 ± 356 | 1751 ± 1097 | 1:1.6 | 0.160 ᵃ |
| **YME1L1** | **NK** | 882 ± 74.9 | 891 ± 83.3 | 878 ± 73.6 | 1:1 | 0.756 |

**Supplementary Table 5. Gene expression analysis.** Normalized counts of gene expression in non-responders and responders as well as gene expression fold change between non-responders and responders. Characteristics of responders and non-responders were compared with a t-test. ᵃ Levene's test is significant (p < .05), suggesting a violation of the equal variance assumption. CTRL, house keeping genes. END, endothelial genes; INFL, inflammation related genes; MYE, myeloid cells related genes; NK, Natural Killer cells related genes; IFNG, interferon gamma related genes; CNI, calcineurin inhibitor toxicity related genes.
